# Ex-vivo validation of spatial gain sonography for the quantification of echo intensity in fascicle-aligned ultrasound images in ten anatomical muscles in *Bos taurus*

**DOI:** 10.1038/s41598-024-53852-0

**Published:** 2024-02-15

**Authors:** Sophie C. Rosahl, Philipp Rauschendorfer, Lukas Arndt, Thomas Voigtmann, Uwe Mittag, Jörn Rittweger

**Affiliations:** 1 Institute of Aerospace Medicine, German Aerospace Center (DLR), Cologne, Germany; 2 Institute of Material Physics in Space, German Aerospace Center (DLR), Cologne, Germany; 3 Institute of Theoretical Physics, Heinrich-Heine-Universität, Düsseldorf, Germany; 4 Department of Pediatrics and Adolescent Medicine, University Hospital Cologne, Cologne, Germany

**Keywords:** Intramuscular connective tissue, Fascia, Musculoskeletal ultrasonography, Spatial gain ultrasonography, B-mode ultrasound, Skeletal muscle, Ultrasonography

## Abstract

This study aimed to validate the concept of spatial gain sonography for quantifying texture-related echo intensity in B-mode ultrasound of skeletal muscle. Fifty-one bovine muscles were scanned postmortem using B-mode ultrasonography at varying fascicle probe angles (FPA). The relationship between mean gray values (MGV) and FPA was fitted with a sinusoidal and a linear function, the slope of which was defined as tilt echo gain (TEG). Macroscopic muscle cross sections were optically analyzed for intramuscular connective tissue (IMCT) content which was plotted against MGV at 0° FPA (MGV_00). MGV peaked at FPA 0°. Sine fits were superior to linear fits (adjusted r^2^-values 0.647 vs. 0.613), especially for larger FPAs. In mixed models, the pennation angle was related to TEG (*P* < 0.001) and MGV_00 (*P* = 0.035). Age was relevant for MGV_00 (*P* < 0.001), but not TEG (*P* > 0.10). The correlation between the IMCT percentage and MGV_00 was significant but weak (*P* = 0.026; adjusted r^2^ = 0.103). The relationship between fascicle probe angle and echo intensity in B-mode ultrasound can be modeled more accurately with a sinusoidal but more practically for clinical use with a linear fit. The peak mean gray value MGV_00 can be used to compare echo intensity across muscles without the bias of pennation angle.

## Introduction

### The role of connective tissue in muscle

Intramuscular connective tissue (IMCT) is essential to the functioning of the muscle. The smallest entity of IMCT is endomysium which surrounds muscle fibrils in interconnected tubular sheaths^1^. Several muscle fibers forming a fascicle are engulfed in perimysium. The whole muscle is surrounded by epimysium. Together, these IMCT entities tightly connect the contractile components of the muscle, organize and uphold the structure of the muscle^2^, transmit forces generated by the myofibrils^3^, and allow for plasticity during growth or injury of the muscle^4^. They also connect to other connective tissues, which have been referred to as ‘fascia’ in the recent past^5^.

### Quantification of intramuscular connective tissue

Evidence suggests that IMCT composition, structure and quantity are altered with age^6–8^, by muscle loading^9,10^, by immobilization^11–14^ and in diseases such as spastic cerebral palsy^15^ and Duchenne muscular dystrophy^16^. Therefore, analyzing IMCT is of diagnostic, therapeutic and prognostic value. Microscopic analyses reveal the structure mainly of endomysium^12,13^ and do not allow for perimysium content assessment. In addition, ex-vivo observations require a muscle biopsy, hence are not feasible for rapid assessment or long-term tracking. A non-destructive, non-invasive, simple, and objective method is desirable for assessment of IMCT quantity and structure. While magnetic resonance imaging studies allow for an overview and volume analysis^6^, a more readily available and more cost-effective method is muscle ultrasonography^17^. Pillen et al. found that the ultrasound echo intensity increases in dystrophic muscle^18^. On physical grounds it can be expected that the structure of IMCT leaves an imprint on echo intensity. Our study aims to explore and exploit this connection further.

### Ultrasonography of intramuscular connective tissue

Ultrasound waves are reflected at surfaces where materials of differing acoustic impedance adjoin^19^. The border between perimysium and muscle fibers comprises one of the main differences in acoustic impedance.

**Figure 1:**
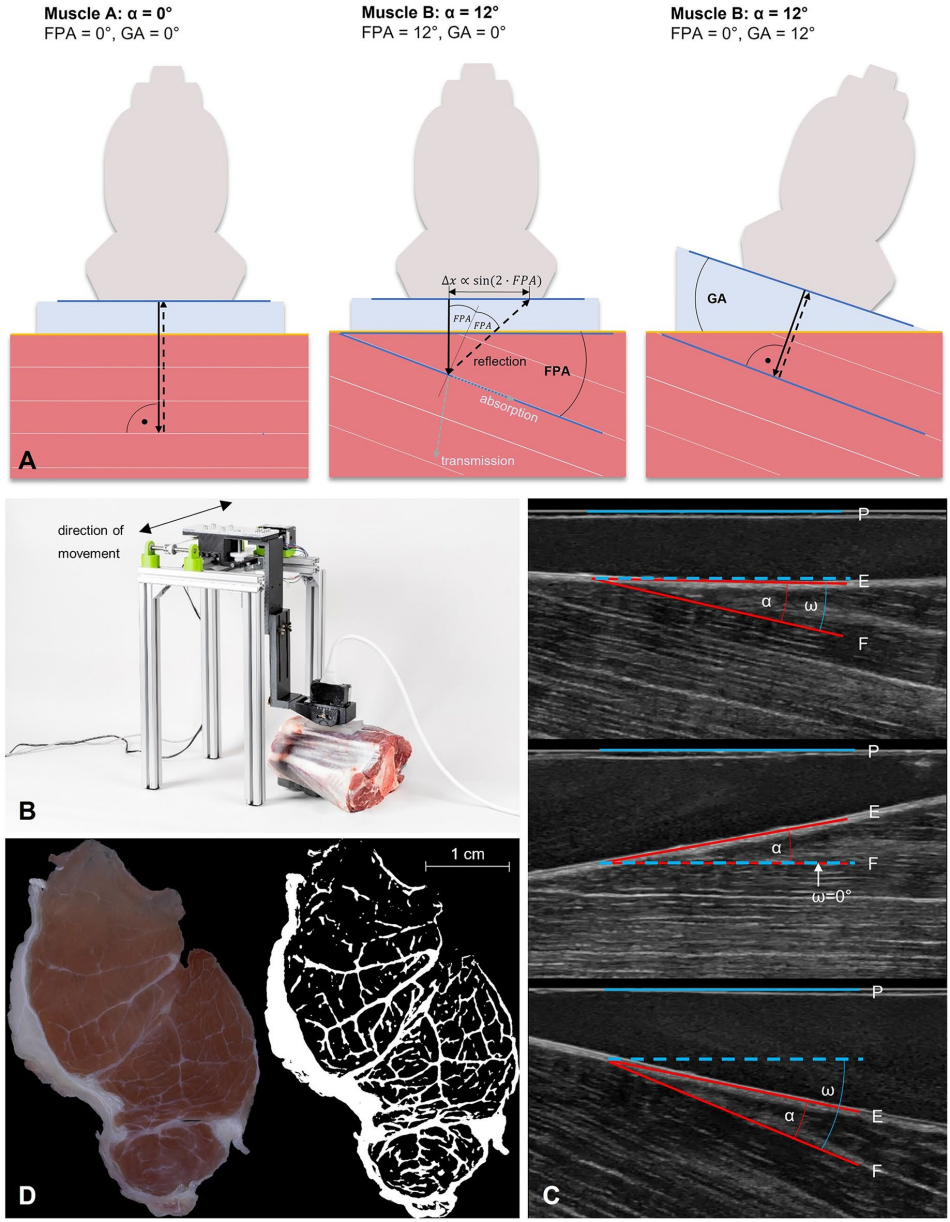
Visualization of ultrasound image acquisition and analysis. (**A**) The fascicle probe angle (FPA) is subject to variation in both the pennation angle (α, middle) and the gel pad angle (GA, right). Only GA can be controlled by the operator, while α stays constant in a resting muscle. The ultrasound gel pad enables angulation affording minimal ultrasound attenuation prior to coupling into the tissue. At a muscle/IMCT interface, reflection, transmission, absorption, and scattering (not indicated) occur and change with the FPA. A change in FPA causes a change in the angle of reflection x =|FPA|, and hence causes the reflected ultrasound ray to be displaced by ∆x ∝ sin(2 $\cdot$|FPA|) in the probe. Thus, a change in FPA will cause all reflected rays within a detector area of fascicle-transverse length ∆x to be reflected away from the probe, and thus the MGV decreases by an amount ∝ ∆x. The image in the middle exemplifies the trigonometric relationship between the probe angle and the amount of ultrasound waves being reflected back to the probe. The echo intensity detected is determined by the function y = β_0_ – (β_1_/2) ∙ sin(2|FPA|) where β_0_ is the mean gray value at 0° FPA (MGV_00) and β_1_ is the tilt echo gain (TEG). (**B**) Set-up for ultrasound image acquisition: The linear probe is held in place by a probe holder that is attached to a robotic axis translating the probe in the shown direction. (**C**) Representative ultrasound images of an infraspinatus muscle scanned with a 0° (left), + 12° (middle) and − 2° (right) gel pad. α is the pennation angle, between fascicle (F) and epimysium (E). ω is the fascicle probe angle (FPA), between fascicle and probe (P). The mean gray value is visibly higher at a smaller FPA (middle < top < bottom). (**D**) Segmentation of macroscopic photo of muscle cross section (left) with *ilastik* (right).

The angle of insonation influences echo intensity (Fig. 1A). The measured reflection is strongest when the ultrasound rays hit the surface perpendicularly and the ultrasound probe is oriented parallel to the surface^19^.

We hypothesize that echo intensity is highest at a fascicle probe angle (FPA) of 0°—when probe and fascicle are parallel (hypothesis 1). We further hypothesize that the relationship between echo intensity and FPA can be mathematically modeled (hypothesis 2). It is also hypothesized that this model is best described by a trigonometric function that can be derived from the laws of specular reflection at the fascicles (hypothesis 3). Lastly, we suggest that this model can be well approximated with a linear relationship between MGV and FPA.

Therefore, we propose that the rate of change in echo intensity per change in FPA will differ for each muscle depending on the architecture of its IMCT. This ratio will be termed tilt echo gain (TEG) here. Notably, a similar technique utilizing angulation in ultrasound has proven useful in detecting experimental inflammation in tendons^20,21^, albeit without mathematical foundation and analysis.

We have conceptualized these above ideas and submitted them for patent (Deutsches Marken- und Patentamt, 10 2019 118 823.7). The present study aimed to validate tilt echo gain by testing our hypotheses 1 to 3.

## Materials and methods

### Provenience and preparation of sample muscles

Ultrasound images of 51 limb and torso muscles of *Bos taurus* were obtained. All animals were female and of either one of the breeds “Rotbunt” and “Schwarzbunt”. Due to the availability of samples only of different age for female and male cattle (only older bulls or young calves) at the abattoir, an un-biased sex comparison was not possible and therefore an analysis of solely female cattle was performed. To emulate in-vivo analyses in humans, post-mortem muscles of domestic cattle were used. The bovine limbs and torso muscles were obtained within 24 h after slaughter from the abattoir *Schlachthof Frenken* in Düren, Germany, where they were cooled at 6 °C, and brought to the lab at the Institute of Aerospace Medicine at the German Aerospace Center (DLR), Cologne, in cooled state. The limbs and muscles were then stored in a refrigerator at 5 °C until their examination zero to two days after retrieval. Before ultrasound scanning was started all muscles were allowed to warm to room temperature (20 °C) to allow for comparability. The animals’ age could be made available by the abattoir only in 12 out of 24 animals.

Ten different muscles were analyzed. From the forelimb, extensor carpi radialis (ECR), flexor carpi radialis (FCR), and extensor digitorum communis (EDC) muscle were scanned. Muscles from the hindlimb included the lateral digital extensor (EDL), fibularis longus (FL), fibularis tertius (FT) muscle and the deep digital flexor muscle with the flexor hallucis longus muscle (FHL) as part of it. Torso muscles included the supraspinatus (SSP), infraspinatus (SCH) and psoas major (PSO) muscle. For ultrasound measurements, the superficial fascia was removed and limb muscles remained attached to the bone.

### Ultrasound scanning procedures

Ultrasound images were obtained using the linear-array probe ML6-15 of a GE LOGIQ S8 ultrasound device (GE Healthcare, Milwaukee, WI, USA) in B-mode with a center frequency of 15 MHz. Ultrasound device settings were kept constant between all measurements, using a scanning depth of 3.5 cm and a gain of 50 dB. Time gain control and CrossXBeam were turned off to avoid corrections to individual images by the ultrasound software.

The limbs or muscles were affixed onto a board. Ultrasound video sequences were obtained with the ultrasound probe placed on the epimysium—which can also be referred to as the aponeurosis—where the muscle fascicles insert. The probe was in longitudinal alignment with the muscle fascicles (Fig. 1A).

Using a 3D-printed probe holder, the probe was mounted to a robotic axis run by an Arduino UNO (*Arduino*, Monza, Italy). The robotic axis allowed for translation of the probe holder by 14.04 mm in a single direction during an ultrasound video sequence (Fig. 1B). Three images from each sequence were used later in order to increase internal validity. The probe holder facilitated to keep the manually adjusted angulation setting of the probe consistent in relation to the epimysium and the longitudinal axis of the fascicles. The robotic axis allowed for consistency of the scanning site between measurements at different angles. An ultrasound gel pad (Vorlaufstrecke SONOKIT soft, 200 × 100 × 40 mm, Co. Sonogel, Bad Camber, Germany; ultrasound velocity $c=1460\frac{{\text{m}}}{{\text{s}}}$, absorption coefficient $a=0.053\frac{dB}{{\text{MHz}}\, {\text{mm}}}$) cut to angles of 0°, 12° and 24° was used for each measurement. Ultrasound video sequences were obtained at 0°, 12°, 24°, -12°, and − 24° probe angle in relation to the epimysium (Fig. 1A).

### Preparation of muscle sections and imaging

Muscles were subsequently cut transversally into three parts equal in length and frozen at − 25 °C. Muscle cross sections of 1 mm thickness were obtained from each of the three parts using a cutting machine (Slicer Master M20, Graef, Arnsberg, Germany). After fixation in a 1:10 37%-formaldehyde/tenfold-PBS buffer solution for four hours, each muscle section was photographed according to a standardized procedure with constant camera and lighting parameters using a digital single-lens mirrorless camera with a macro lens. These images were then segmented via thresholding using the machine learning software *ilastik: Interactive Learning and Segmentation Toolkit* (ilastik Team, 2011) that was initially fed with data from manual segmentation differentiating between IMCT and muscle visually. The images were then evaluated for IMCT content using the percentages of pixels above and below that threshold determined by the machine learning algorithm for all images (Fig. 1D).

### Image processing

A three-step custom-made Python algorithm (Python 3, http://www.python.org), QuantICUS (Quantification of Intramuscular Connective tissue with Ultrasound) in version 1.0, was developed to extract single images from the ultrasound video sequences at the same locations in each muscle and to determine the pennation angle α, FPA (Fig. 1c) and mean gray value (MGV) in a region of interest (ROI) of the same rectangular size and orientation in each image (for further details see Supplementary Material “Image Processing: Calculation of Pennation Angle and Fascicle Probe Angle”).

### Data processing

Output data from the QuantICUS tool were further processed and analyzed with R (http://www.r-project.org) in its version 4.2.2 by merging data obtained from separate muscles into one database. Homogeneity of frames within sweeps was verified by statistically testing for the effect of frame number (i.e. of probe translocation) upon epimysium angle and MGV, which both were found to be non-significant (linear mixed effect models from R-package ‘nlme’, all *P* > 0.20). Accordingly, all frames were included in the analysis.

Next, we assessed the relationship between FPA and MGV, hypothesizing that a trigonometric function would serve this purpose (s. Figure 1A). Fitting was effectuated with the basic R-function ‘lm’, with a prior sine-transformation of the independent variable (y = β_0_ − (β_1_/2) ∙ sin(2x) where x is the absolute value of FPA). For comparison and simplicity reasons, we also tried a linear fit (y = β_0_ − β_1_ · x) which can be derived from the trigonometric function for smaller angles when sin(2x) approximates 2x. To compare between both fitting results, we used residual plots and quantile–quantile plots to judge residual deviation from normality, and also Akaike’s information criterion.

To finally obtain spatial gain sonography results, we then fitted a linear relationship between FPA and MGV for all individual muscle specimens, in order to arrive at estimates for the expected gray value at FPA 0° (MGV_00 = β_0_). Tilt echo gain (TEG) was then computed as 100 * β_1_/ β_0_, to give the % change in MGV per FPA change (s. Table 2).

### Statistical analyses

Correlations between TEG values were performed with the R-function ‘lm’ after visual inspection of the plots. The following criteria were adopted to interpret the magnitude of the correlations: *r* < 0.1, trivial; 0.1 < *r* ≤ 0.3, small; 0.3 < *r* ≤ 0.5, moderate; 0.5 < *r* ≤ 0.7, large; 0.7 < *r* ≤ 0.9, very large; and *r* > 0.9, almost perfect^20^.

Linear mixed effect models were run with the R-function ‘lme’ to compare differences in pennation angle α with ‘Muscle’ as fixed factor and animal ID as random effect. Similarly, differences in MGV_00, TEG, and in ROICy (y-coordinate of the region of interest in the ultrasound scans) were tested with setting Muscle and α as fixed effects. Separate models were run, once excluding Age and once including Age. From the mixed models, we extracted β coefficients, i.e. the offset and slope for each fixed factor, as well as the contribution from fixed effect variables towards the overall r^2^-value, using the function ‘rsq.lmm’ from the library ‘rsq’. All models were scrutinized with residual plots and quantile–quantile plots, and residuals were found to be well-behaved for all models.

Data are presented as means (standard deviation) if not stated otherwise. The level of significance was set to 0.05.

## Results

Animals were between 3.34 and 8.22 years old (mean 5.90 years). There were no missing datasets from the image processing, and no data were excluded during data processing, so that all data could be subjected to statistical analysis. Table 1 gives an overview of the number of tested muscle specimens.

**Table 1:** Overview of specimens tested.

Muscle	A0	B8	C0	C5	D8	F1	G2	H0	H7	J1	J6	K9	N6	O2	P0	P3	R6	S7	S9	T8	U1	V5	V9	Y8	**Sum**
ecr	0	0	0	1	1	0	0	0	1	0	1	0	1	1	1	0	0	0	1	0	0	0	0	0	**8**
edc	0	0	0	1	1	0	0	0	1	0	1	1	1	1	1	0	0	0	1	1	0	0	0	0	**10**
edl	0	0	0	0	0	0	0	0	0	1	0	0	0	0	0	0	0	0	0	0	0	0	0	0	**1**
fcr	0	0	0	1	1	0	0	0	1	0	1	1	1	1	1	0	0	0	1	1	0	0	0	0	**10**
fhl	0	0	0	0	0	0	0	1	0	0	0	0	0	0	0	1	1	0	0	0	0	0	1	0	**4**
fl	0	0	0	0	0	0	0	1	0	1	0	0	0	0	0	1	1	0	0	0	0	0	1	0	**5**
ft	0	0	0	0	0	0	0	1	0	0	0	0	0	0	0	1	1	0	0	0	0	0	1	0	**4**
pso	0	0	1	0	0	0	0	0	0	0	0	0	0	0	0	0	0	1	0	0	1	0	0	0	**3**
sch	1	0	0	0	0	1	1	0	0	0	0	0	0	0	0	0	0	0	0	0	0	0	0	0	**3**
ssp	0	1	0	0	0	0	0	0	0	0	0	0	0	0	0	0	0	0	0	0	0	1	0	1	**3**
Sum	**1**	**1**	**1**	**3**	**3**	**1**	**1**	**3**	**3**	**2**	**3**	**2**	**3**	**3**	**3**	**3**	**3**	**1**	**3**	**2**	**1**	**1**	**3**	**1**	**51**
Age					6.08			3.34	8.22	5.52	6.34		6.42	4.99		5.11	4.43		8.22	6.97			5.11		

Highlighted in bold is the sum of analyzed muscles per animal in each column and the sum of different anatomical muscles analyzed overall in each row.

**Figure 2:**
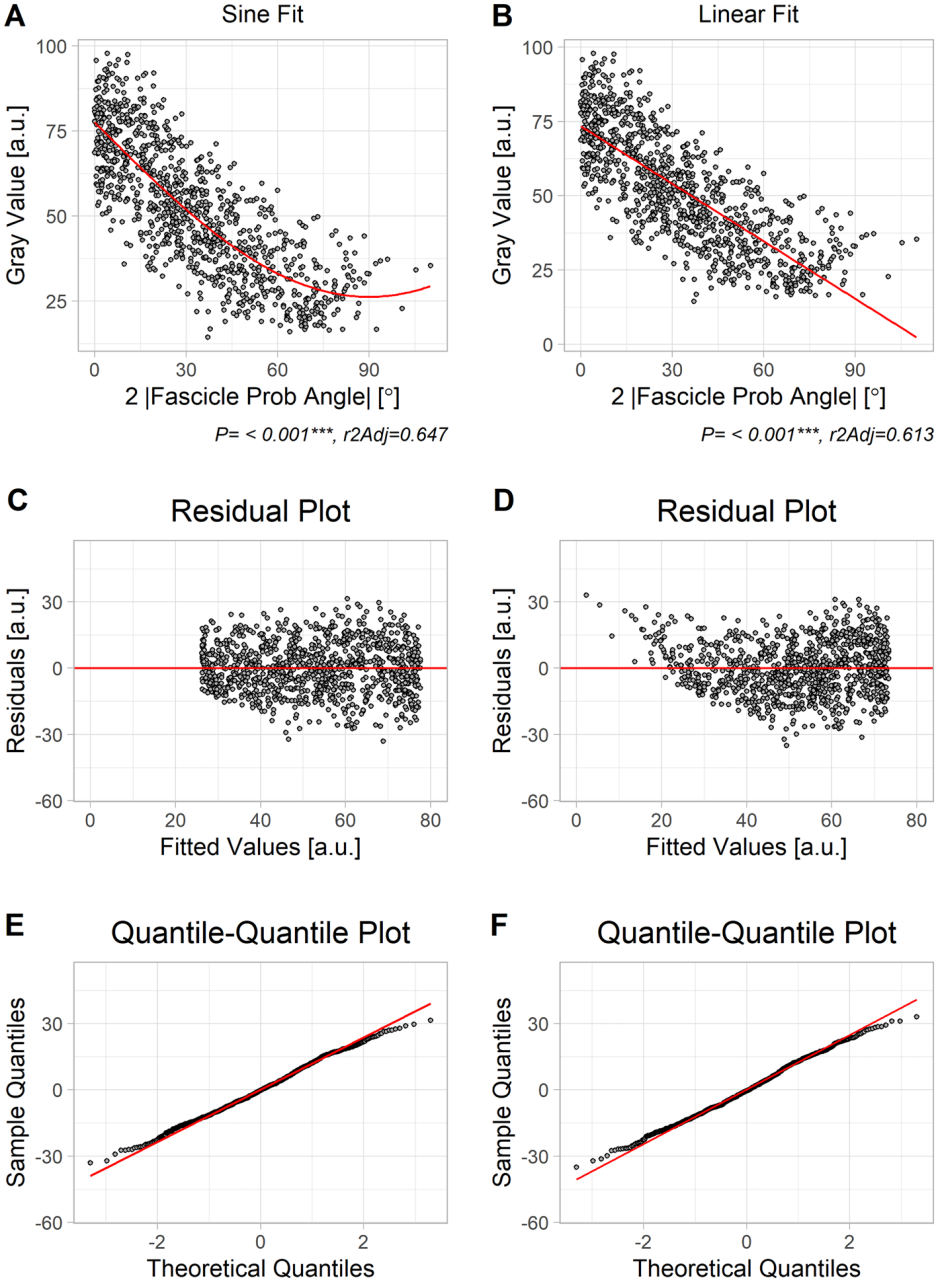
Comparing results for fitting a sine function (left column) *versus* a linear function (right column) for the grand ensemble of all data. Note that both fits were performed with absolute FPA as independent variable. (**A**, **B**) Scatter plot of raw data with fitted function curves. As can be seen, gray values peaked at FPA = 0°. (**C**, **D**) Residuals (i.e. vertical distance of data point from fitted line in upper row) plotted vs. fitted values (i.e. y-coordinate of fitted curve in A and B, upper row). As can be seen, residuals at extreme ends of x-axis deviate more from 0 in D than in C. (**E**, **F**) Observed quantiles plotted *versus* expected quantiles. A straight line would indicate normal distribution of residuals. Although residuals from both fittings seem to be reasonable, they seem slightly closer to normal distribution at extreme ends for the sinusoidal fit.

**Figure 3:**
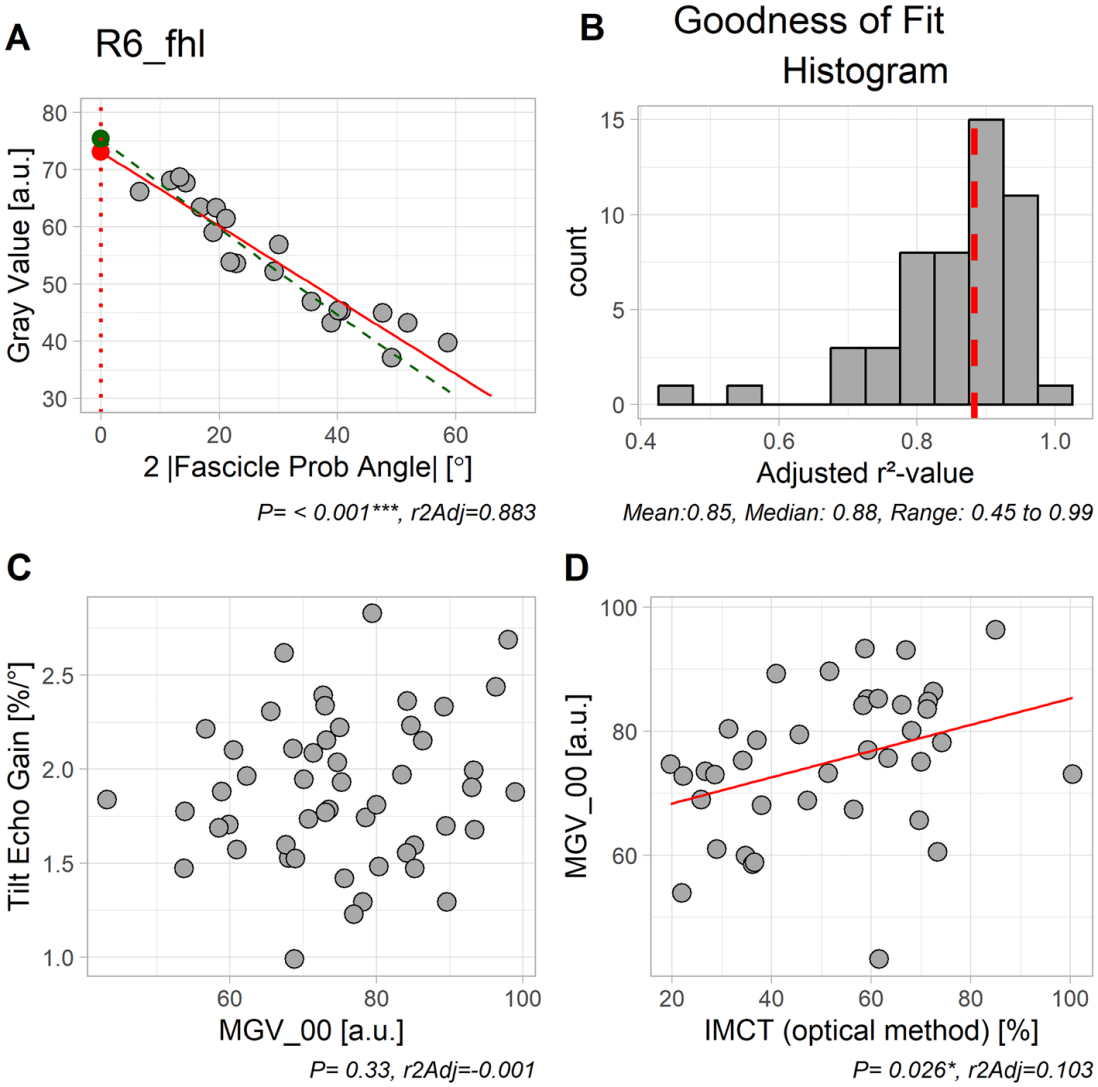
Illustration of spatial gain parameters. (**A**) The green line indicates the sinusoidal fit and the red line the linear fit. The green and red circle represent the predicted MGV_00 (gray value at FPA 0°) for each fit. Estimates for MGV_00 were directly derived from the fitted line at x-value 0° and the negative slope was defined as tilt echo gain in percent MGV_00 per degree (TEG). For this plot, we used data from fhl muscle in animal R6, since the adjusted r^2^-value for that specimen coincided with the median of all adjusted r^2^-values. (**B**) Distribution of all adjusted r^2^-values, with the median marked as vertical dashed line in red. (**C**) Correlation between TEG and MGV_00 was very weak. (**D**) Correlation between MGV_00 and IMCT ratio measured optically in cross sections was significant, but weak.

MGV peaked for FPA = 0° (Fig. 2A,B). When comparing the results for fitting a sine function y = β_0_ – (β_1_/2)· sin(2|FPA|) (Fig. 2A) with results for a linear fitting y = β_0_ − β_1_ · |FPA| (Fig. 2B), the former was superior both in terms of adjusted r^2^-values (0.647 vs. 0.613) and Akaike’s information criterion (7846 vs. 7942). For greater values of FPA residuals were larger for the linear plot (Fig. 2D), thus with slightly greater deviation from normal distribution (Fig. 2F) than for the sine fit (Fig. 2C,E). This demonstrates the correctness of the assumed sine function, but also that the function can conveniently be linearized as long as the range of observation is not too far from 0°. Therefore, the spatial gain sonography parameters were calculated with the linear fit (Fig. 3A), as this will be more practicable in the clinical setting. The adjusted r^2^-values were > 0.7 for 48 out of 51 specimens (see Fig. 3B). TEG was found to be entirely unrelated to MGV_00 (*P* = 0.33, Fig. 3C) (Table 2).

**Table 2:** Descriptive statistics (given as means and their standard deviation in brackets) for the different anatomical muscles tested, and results of testing with mixed effect models and Dunn’s post hoc test, using the entire data set and not including Age into the models.

Variable	*pso*	*edc*	*ecr*	*ft*	*fcr*	*fhl*	*sch*	*fl*	*edl*	*ssp*	*P.α*	*P. Muscle*	*post-hoc*
*N*	3	10	8	4	10	4	3	5	1	3	–	–	–
*α*	11.1 (0.5)	7.4 (5.2)	13.1 (3.9)	17 (6.9)	11.6 (4.9)	10.2 (2.3)	15.2 (4.9)	10 (10.3)	14.3 (NA)	1.6 (0.4)	–	0.043*	ft—ssp (*P* = 0.017)
*MGV_00*	66.8 (5.7)	68.9 (13.7)	72.1 (13.1)	72.3 (4.7)	72.5 (10.9)	75.4 (12.6)	83.1 (11.2)	84.8 (7.1)	93.1 (NA)	94.1 (7.7)	0.035*	0.041*	No siginficant post-hoc findings
*TEG*	2.1 (0.01)	1.98 (0.35)	1.68 (0.27)	1.31 (0.26)	2.16 (0.31)	1.89 (0.21)	1.65 (0.33)	1.9 (0.49)	1.9 (NA)	2.01 (0.62)	< 0.001***	0.01**	ft—fcr (*P* = 0.019)
*ROICy*	410.14 (16.69)	449.67 (61.02)	420.93 (39.68)	380.42 (13.97)	419.31 (47.01)	402.23 (42.72)	414.67 (37.65)	438.53 (41.78)	431.82 (NA)	439.14 (43.38)	0.17	0.24	–

**Figure 4:**
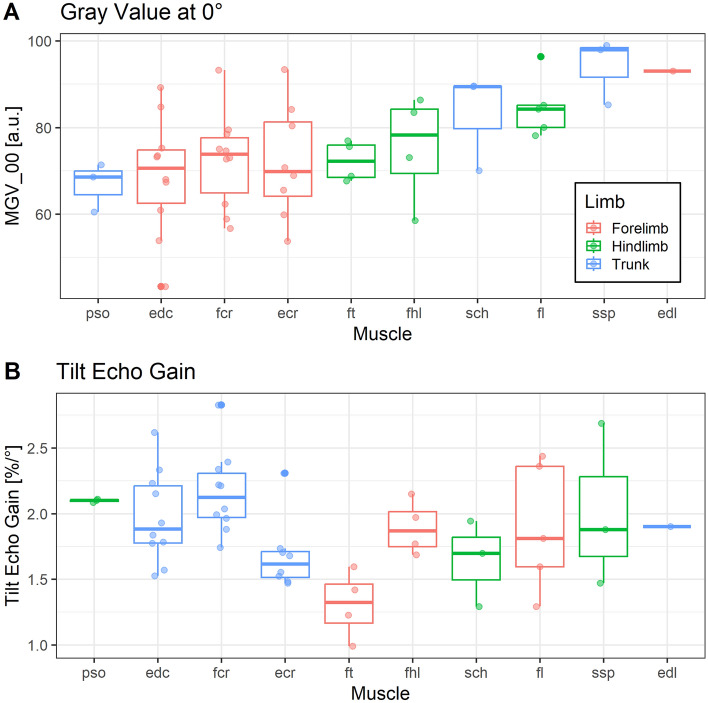
Box plots for MGV_00 and TEG across all analyzed muscles, shown by identical anatomical muscles (x axis) and grouped by forelimb, hindlimb and trunk muscles via color. (**A**) Forelimb muscles (edc, ecr, fcr, edl), hindlimb muscles (ft, fhl, fl) and trunk muscles (pso, sch, ssp) can hardly be distinguished by MGV_00, rather, differences exist between each muscle. (**B**) TEG is distributed differently across muscles compared with MGV_00.

Visual inspection of MGV_00 and TEG suggested muscle-specific variation in MGV_00 and TEG across the different anatomical muscles (Fig. 4) That impression was confirmed by statistical testing with mixed effect models, which revealed muscle-related differences with regards to α (*P* = 0.043), MGV_00 (*P* = 0.041) and TEG (*P* = 0.01). The pennation angle α was also related to TEG (*P* < 0.001) and MGV_00 (*P* = 0.035) across Muscle. No effects of Muscle or α were observed for ROICy (both *P* > 0.1), suggesting that variation in scanning depth does not explain the former findings. When including age as fixed factor into these mixed effect models, it was significant for MGV_00 (*P* < 0.001) but not for TEG (*P* > 0.10). Of note, Muscle remained significant for MGV_00 and TEG after including age into the mixed models, and the amount of variation that was explained by fixed factors (r^2^ fixed in Table 3) increased. The percentage of IMCT in a muscle cross section measured optically significantly affected MGV_00 but the amount of variance accounted for by the regression was low (r^2^ = 0.103).

**Table 3:** Comparison of linear mixed model results with excluding or including Age as fixed effect.

Variable	Age excluded				Age included				
	Offset	P (Muscle)	β (α)	r^2^ fixed	Offset	P (Muscle)	β (α)	β (Age)	r^2^ fixed
MGV_00	74.6*** (60.8 to 88.4)	0.041	− 0.7* (− 1.29 to − 0.11)	0.43	27.6*** (7.7 to 47.5)	0.013	− 0.61* (− 1.12 to − 0.1)	6.5*** (3.8 to 9.2)	0.68
TEG	2.48*** (2.1 to 2.86)	0.01	− 0.034*** (− 0.051 to − 0.017)	0.55	1.99*** (1.3 to 2.68)	0.007	− 0.032** (− 0.049 to − 0.015)	0.04 (− 0.05 to 0.13)	0.63

Beta denotes the regression coefficient, and r^2^ fixed the contribution from fixed factors to the overall r^2^ value. Values for Offset and Beta are given as means (95% confidence interval). Asterisks denote significance as follows: *P < 0.05, ***P < 0.001.

## Discussion

All three initial hypotheses were confirmed: Echo intensity is generally peaking at a fascicle probe angle of 0° (hypothesis 1). Echo intensity decreases with increasing deviation from a 0° fascicle probe angle which can be modeled with a sinusoidal function and approximated with a linear function (hypothesis 2 and 3).

TEG*,* i.e. the slope of this linear function for each muscle, and MGV_00 did not show a strong correlation. MGV_00 can be seen as an extensive measure describing the echo intensity (quantity) of echo-reflecting texture within the muscle tissue. As a second, independent descriptor, TEG can be regarded as a more ‘qualitative’ variable that probably assesses the order of ‘directionality’ of the tissue-textures. MGV_00 and TEG were differently expressed between different anatomical muscles. The pennation angle was related to MGV_00 and TEG across muscle groups. Age increased the amount of variance explained by anatomically different muscles and pennation angle in the mixed models. Increased echo intensity at FPA 0° in ultrasound correlated with an increased percentage of IMCT in a macroscopic muscle cross section when measured optically, suggesting that echo intensity could at least in part reflect the amount of IMCT.

### Tilt echo gain fit

Echo intensity was found to be dependent on fascicle probe angle, with the maximum echo intensity measured at 0° FPA. We originally assumed that a sinusoidal function would prove a better fit than a linear function due to trigonometric laws underlying the physics of reflection, and that was indeed the case. Other factors such as differences in acoustic impedance between muscles may influence echo intensity but the angle-dependency clearly dominates, hence a trigonometric regression could be derived. Interestingly however, a linear function showed almost equal performance, in particular at smaller fascicle probe angles. This can be explained by the fact that deviations of y = 2 × from y = sin(2x) amount to approximately 10% at an angle of 20°. For an angle of 0° (MGV_00) which was of particular interest in this study, results obtained from a linear fit compared to the trigonometric fit showed almost no difference. A linear variation of the intensity with FPA approaching zero can be taken as the most generic model-free expectation, und thus suggests itself as a robust analysis method for small enough FPAs. In consequence, we opted to work with the linear function for practicality and suggest that use of this function and clinical ultrasound measurements be restricted to an FPA range between − 30° and 30°.

It should be noted though that the ultrasound software might have affected the gray values in the images via post-processing. It was ensured that time gain control was kept constant at all levels, and that other correction features were turned off to avoid intrinsic correction of the images. Nonetheless, insight into the complete ultrasound post-processing algorithm is probably needed to fully comprehend and exclude the possibility of interference with the acquired data.

### Comparison of different anatomical muscles

The muscle specimens tested in this study yielded systematic variation with regard to MGV_00 and TEG across the different anatomical muscles. This was more evident for TEG than for MGV_00, as the latter failed to yield any significant post hoc results. For TEG, a significant post hoc difference was found between fibularis tertius muscle (hindlimb) and the supraspinatus muscle (torso). However, there was no general trend for trunk, hindlimb or forelimb muscles.

It should be considered that this study was not designed to find IMCT differences across anatomical muscles, but rather included a variety of different muscles to validate the concept of spatial gain sonography. Moreover, two factors need to be considered here. First, muscles from different breeds of *Bos taurus* were analyzed and literature suggests that muscle architecture varies between different breeds^22,23^. However, that group also found that the variation in the density of slow muscles fibers had greater variation within the muscle than across breeds which relativizes the problem of different breeds but reveals another obstacle for the comparison between muscles. The scanning site was not always the exact same in every anatomically equal muscle. Rather, attention was given to choosing an area with parallel fascicles without blood vessels and sufficient muscle thickness to scan across all angles. In order to make anatomical observations across different muscles, the muscle structure varying within a muscle needs to be considered^24,25^.

It is apparent from Fig. 4, however, that variation in TEG and in MGV_00 was different across different anatomical muscles, further corroborating that these two measures are independent and convey diverging information.

### Influence of pennation angle on spatial gain parameters

We also observed an effect of pennation angle α on spatial gain sonography parameters independent of FPA. Muscles are pennated in order to increase force per unit muscle mass^26^. Because muscles differ in their function and size, their pennation angles vary likewise as observed in this study. The pennation angle might therefore depend on the function of the specific muscle, e.g. load-bearing antigravity muscles versus non-antigravity muscles, which in turn could influence IMCT architecture and hence spatial gain parameters.

### Influence of age on spatial gain parameters

Age was significant for MGV_00 when included as a fixed factor into mixed models. Other studies have confirmed increased echo intensity at higher age^27–29^. However, as clearly demonstrated in this study, echo intensity is subject to fascicle probe angle changes. Therefore, and as much as pennation angle systematically decreases with aging^30–32^, reporting age-related effects without adjusting echo intensity for fascicle probe angle, surely has potential to over-estimate age and immobilization-related changes. In that sense, our new parameter MGV_00 can be regarded as a standardized and more robust version of ‘echo intensity’ as it was used in literature for characterizing perimysium in skeletal muscle.

### Correction for pennation angle in echo intensity measurements

Former clinical studies have suggested that increases in muscular echo intensity can be utilized in the diagnostic approach to muscle atrophy and dystrophy^33,34^. However, dystrophy and atrophy have a bearing on pennation angle. Whilst pennation angle decreases with immobilization-related atrophy^35^ it was found to be increased in Duchenne muscular dystrophy^36^. Again, such effects will lead to bias, and to systematic under- or over-estimation of skeletal muscle echo intensity, unless adjustment is performed. One group researching neuromuscular disorders in children visually corrected for pennation angle by adjusting the probe over the muscle until the echo intensity was highest^37,38^. Another study even measured echo intensity alteration with a probe tilt up to 6° in both directions in relation to the skin and proposed that the operator shall minimize the probe tilt during muscle examination^39^. While both strategies are in line with the presented evidence, they are less quantitative than our approach, raising issues of intra- and inter-operator reproducibility. Moreover, whilst those proposed ‘work-arounds’ for echo intensity could be regarded as precursors for MGV_00, we are not aware of any former attempt to model and assess TEG functions.

### Reflection of perimysium content in echo intensity

Upon optical analysis of muscle cross sections, higher IMCT percentages were measured for higher values of MGV_00 from ultrasound analysis. This finding is in line with a previous study that showed that histological fibrous tissue content in dogs with muscular dystrophy linearly correlates with echo intensities in the respective muscles^18^. As opposed to that study, we took samples from healthy bovine muscles and analyzed macroscopic cross sections of the whole muscle instead of microscopic images. We presumed that the perimysium measured in ultrasound would be better represented by these large sections as perimysium is too vast to be estimated reliably in one microscopic field. This approach in turn relies on the assumption that solely perimysium, and not endomysium, is reflected in ultrasound echo intensity measurements. Axial resolution, the resolution longitudinal to the ultrasound beam, depends on the length of an ultrasound pulse and the wavelength. A smaller wavelength will also lead to a shorter pulse length thus allowing for better discrimination between objects that are closer together. An ultrasound wave with a frequency of 15 MHz traveling through bovine muscle, in which the average speed of sound is 1580 m/s^40^, has a wavelength of 105 μm. The best axial resolution that can be achieved is half the pulse length^41^. Under optimal conditions, if the pulse length is only one wavelength and hence axial resolution is 52.5 μm, bovine perimysium with a thickness between 30 and 120 μm will contribute to an echo alteration^42,43^. However, endomysium with a thickness of 5 to 15 μm depending on the muscle^43^ is probably not represented in current B-Mode ultrasound images and at most contributes to scattering of ultrasound waves which occurs when the wavelength is considerably greater than the size of the encountered object^44^.

In addition, in this study we were careful not to include planes with visible adipose tissue, blood vessels or nerves that might cause increases in echo intensity. Nonetheless, for in-vivo studies this is sometimes unavoidable and it therefore remains a matter of discussion whether echo intensity truly reflects the amount of IMCT in a muscle^45,46^.

### Discussion of implementation in the clinical setting

Beam steering is one tool already implemented into ultrasound software which would allow for implementation of spatial gain sonography into clinical practice. The clinician would have to obtain an ultrasound image of a muscle longitudinally with no angulation first. Second, an image of the exact same location using beam steering is needed. In one of these images, the pennation angle would need to be measured, which is possible on most ultrasound devices or can be measured using computer software. Using the linear function, MGV_00 would result from the regression fit of mean gray value and fascicle probe angle. The measurement can be repeated at any given time point and MGV_00 tracked over time for a muscle echo intensity evaluation without the influence of a potentially changing layer of subcutaneous fat or an altered muscle volume in patients.

### Limitations

Although our study did find significant effects by anatomical muscle in age and pennation angle, the study was not designed to find such effects. Therefore, the interpretation of these effects must remain on a somewhat speculative level. It should also be noted that our photographic image analysis of the perimysium assessed quantity, but not structural or textural information. However, conducting such an assessment would constitute a considerable undertaking, and we therefore decided to demonstrate the viability of spatial gain sonography in principle before making larger investments into potentially moot and certainly costly perimysium assessments.

Also, one needs to consider that our specimens stem from older female cattle only, and did not contain younger or very old animals, and also no male cattle. The next step will be a larger human study to validate our novel approach, and to further explore the effects of age, sex, immobilization and exercise training.

The potential effects on echo intensity from using an ultrasound gel pad should also be discussed. Different scanning angles required gel pads of varying thickness, which lead to a varying amount of sound wave energy absorption in each angle setting. This may have biased our results, which is placed into a relative context again by (1) the absorption coefficient of the gel pad being only half that of muscle (0.053 vs. 0.11 $\frac{dB}{{\text{MHz}} \, {\text{mm}}}$ respectively^47^) and (2) the proportions of the gel pad: the gel pad for 0° was 1 cm in thickness while the gel pad for 12° was 0 cm on one end and 2 cm on the other allowing for some amount of averaging out of the mentioned distortion. Due to these circumstances, a complex calculation of the minor effects of the gel pad on echo intensity in each image was not included in this analysis.

Another limitation arises from the fact that we used a commercial ultrasound scanner, and that the image processing therefore contains elements unknown to us^48^. For example, ultrasound images may be displayed using a logarithmic scale instead of linear which would influence the model. However, this device-internal information could not be verified and therefore remains a limitation of the study which can only be circumnavigated using hard to obtain raw ultrasound data in future studies. For example, there is a possibility that brightness is adjusted for depth by the machine, and that the observed effects by pennation angle could be a by-product of our study scanning deeper tissue portions for muscles with greater pennation angle^49,50^. To rule that possibility out, we examined whether pennation angle was related to scanning depth (ROICy). This was not the case, which enhances our confidence in the results related to pennation angle, as well as the viability of spatial gain sonography in general.

## Conclusions

The present study has validated the concept of spatial gain sonography in principle by demonstrating that echo intensity peaks when the probe is parallel to skeletal muscle fascicles. Hereby, we can derive MGV_00 as an estimate of perimysium content that is not biased by concomitant variation in pennation angle. Our method can also work in cases where the ultrasound cannot be aligned parallel to the fascicle orientation, e.g. in muscles with large pennation angle or with an anatomical axis that is oblique to the skin. For these reasons MGV_00 constitutes an improvement of the currently used ‘echo intensity’ that could be readily adopted for making medical diagnoses.

Perhaps even more importantly, we showed that through introduction of TEG as a new variable, another tissue property can be captured that assesses systematic and meaningful effects that are largely independent of MGV_00. Future fundamental research will be required to develop a better understanding of what this variable reflects where it is clinically relevant. If the outcome of that future research demonstrates viability of the spatial gain approach to yield clinically relevant information, then the scanning analysis procedures would have to be standardized for clinical usage, e.g. by replacing the gel pads with a more time-efficient method. Eventually, automated computation of TEG and MGV_00 into the scanner software would further facilitate the applicability.

### Supplementary Information

Supplementary Information.

## Data Availability

All data analyzed during this study are available from the corresponding author on reasonable request.

## Abbreviations

α: Pennation angle
DLR: Deutsches Zentrum für Luft- und Raumfahrt (German Aerospace Center)
ECR: Extensor carpi radialis muscle
EDC: Extensor digitorum communis muscle
EDL: Lateral digital extensor muscle
FCR: Flexor carpi radialis muscle
FHL: Flexor hallucis longus muscle
FL: Fibularis longus muscle
FPA: Fascicle probe angle
FT: Fibularis tertius muscle
GA: Gel pad angle
IMCT: Intramuscular connective tissue
MGV: Mean gray values
MGV_00: Mean gray value at 0° fascicle probe angle
MRI: Magnetic resonance imaging
PSO: Psoas major muscle
ROI: Region of interest in ultrasound images
ROICy: Scanning depth or y-coordinate of the region of interest in ultrasound images
SCH: Infraspinatus muscle
SSP: Supraspinatus muscle
TEG: Tilt echo gain

## References

[1] Sleboda DA, Stover KK, Roberts TJ (2020). Diversity of extracellular matrix morphology in vertebrate skeletal muscle. J. Morphol..

[2] Purslow PP (2002). The structure and functional significance of variations in the connective tissue within muscle. Comp. Biochem. Physiol. Part A Mol. Integr. Physiol..

[3] Ramaswamy KS (2011). Lateral transmission of force is impaired in skeletal muscles of dystrophic mice and very old rats. J. Physiol..

[4] Purslow PP (2010). Muscle fascia and force transmission. J. Bodywork Mov. Therap.

[5] Wilke J, Krause F, Vogt L, Banzer W (2016). What is evidence-based about myofascial chains: A systematic review. Arch. Phys. Med. Rehabil..

[6] Csapo R, Malis V, Sinha U, Du J, Sinha S (2014). Age-associated differences in triceps surae muscle composition and strength—An MRI-based cross-sectional comparison of contractile, adipose and connective tissue. BMC Musculoskeletal Disord..

[7] Fede C (2022). The effects of aging on the intramuscular connective tissue. Int. J. Mol. Sci..

[8] Wojtysiak D (2013). Effect of age on structural properties of intramuscular connective tissue, muscle fibre, collagen content and meat tenderness in pig longissimus lumborum muscle. Folia Biol. (Krakow).

[9] Williams PE, Goldspink G (1981). Connective tissue changes in surgically overloaded muscle. Cell Tissue Res..

[10] Mendias CL, Schwartz AJ, Grekin JA, Gumucio JP, Sugg KB (2017). Changes in muscle fiber contractility and extracellular matrix production during skeletal muscle hypertrophy. J. Appl. Physiol. (Bethesda, Md.: 1985).

[11] Haus JM, Carrithers JA, Carroll CC, Tesch PA, Trappe TA (2007). Contractile and connective tissue protein content of human skeletal muscle: Effects of 35 and 90 days of simulated microgravity and exercise countermeasures. Am. J. Physiol. Regul. Integr. Comp. Physiol..

[12] Järvinen TAH, Jozsa L, Kannus P, Jarvinen TLN, Jarvinen M (2002). Organization and distribution of intramuscular connective tissue in normal and immobilized skeletal muscles. An immunohistochemical, polarization and scanning electron microscopic study. J. Muscle Res. Cell Motil..

[13] Mayer WP, Baptista JDS, Oliveira F, de Mori M, Liberti EA (2021). Consequences of ankle joint immobilisation: Insights from a morphometric analysis about fibre typification, intramuscular connective tissue, and muscle spindle in rats. Histochem. Cell Biol..

[14] Williams PE, Goldspink G (1984). Connective tissue changes in immobilised muscle. J. Anat..

[15] Booth CM, Cortina-Borja MJF, Theologis TN (2001). Collagen accumulation in muscles of children with cerebral palsy and correlation with severity of spasticity. Dev. Med. Child Neurol..

[16] Desguerre I (2009). Endomysial fibrosis in Duchenne muscular dystrophy: A marker of poor outcome associated with macrophage alternative activation. J. Neuropathol. Exp. Neurol..

[17] Lee JC, Healy J (2004). Sonography of lower limb muscle injury. AJR. Am. J. Roentgenol..

[18] Pillen S (2009). Skeletal muscle ultrasound: correlation between fibrous tissue and echo intensity. Ultrasound Med. Boil..

[19] Li J, Ming-Der Chow R, Vadivelu N, Kaye AD (2021). Ultrasound Fundamentals.

[20] Hinkle D, Wiersma W, Jurs SG (2003). Applied Statistics for the Behavioral Sciences.

[21] Lehtinen A, Bondestam S, Taavitsainen M (1994). Use of angulation in the detection of tendinitis with US. Eur. J. Radiol..

[22] Albrecht E, Teuscher F, Ender K, Wegner J (2006). Growth- and breed-related changes of muscle bundle structure in cattle. J. Anim. Sci..

[23] Albrecht E, Lembcke C, Wegner J, Maak S (2013). Prenatal muscle fiber development and bundle structure in beef and dairy cattle. J. Anim. Sci..

[24] Oranchuk DJ, Stock MS, Nelson AR, Storey AG, Cronin JB (2020). Variability of regional quadriceps echo intensity in active young men with and without subcutaneous fat correction. Appl. Physiol. Nutr. Metabol..

[25] Rabello R (2019). Echo intensity reliability between two rectus femoris probe sites. Ultrasound (Leeds, England).

[26] Roberts TJ (2019). The multi-scale, three-dimensional nature of skeletal muscle contraction. Physiology (Bethesda, Md.).

[27] Ryan ED (2015). Pennation angle does not influence the age-related differences in echo intensity of the medial gastrocnemius. Ultrasound Med. Boil..

[28] Ota M, Ikezoe T, Kato T, Tateuchi H, Ichihashi N (2020). Age-related changes in muscle thickness and echo intensity of trunk muscles in healthy women: Comparison of 20–60s age groups. Eur. J. Appl. Physiol..

[29] Kobayashi K (2022). Effect of age on shear modulus, muscle thickness, echo intensity of the upper limb, lower limb, and trunk muscles in healthy women. Eur. J. Appl. Physiol..

[30] Morse CI, Thom JM, Reeves ND, Birch KM, Narici MV (2005). In vivo physiological cross-sectional area and specific force are reduced in the gastrocnemius of elderly men. J. Appl. Physiol. (Bethesda, Md.: 1985).

[31] Strasser EM, Draskovits T, Praschak M, Quittan M, Graf A (2013). Association between ultrasound measurements of muscle thickness, pennation angle, echogenicity and skeletal muscle strength in the elderly. Age (Dordrecht, Netherlands).

[32] Jacob I, Johnson MI, Jones G, Jones A, Francis P (2022). Age-related differences of vastus lateralis muscle morphology, contractile properties, upper body grip strength and lower extremity functional capability in healthy adults aged 18 to 70 years. BMC Geriatr..

[33] Pillen S (2007). Quantitative skeletal muscle ultrasound: Diagnostic value in childhood neuromuscular disease. Neuromuscul. Disord..

[34] MacLennan RJ (2020). Declines in skeletal muscle quality versus size following two weeks of knee joint immobilization. PeerJ.

[35] Seynnes OR, Maganaris CN, de Boer MD, Di Prampero PE, Narici MV (2008). Early structural adaptations to unloading in the human calf muscles. Acta Physiologica (Oxford, England).

[36] Bulut N (2022). Ultrasonographic assessment of lower limb muscle architecture in children with early-stage Duchenne muscular dystrophy. Arquivos de neuro-psiquiatria.

[37] Scholten RR, Pillen S, Verrips A, Zwarts MJ (2003). Quantitative ultrasonography of skeletal muscles in children: Normal values. Muscle Nerve.

[38] Pillen S (2006). Skeletal muscle ultrasonography: Visual versus quantitative evaluation. Ultrasound Med. Biol..

[39] Dankel SJ (2020). The impact of ultrasound probe tilt on muscle thickness and echo-intensity: A cross-sectional study. J. Clin. Densitom..

[40] Ludwig GD (1950). The velocity of sound through tissues and the acoustic impedance of tissues. J. Accoust. Soc. Am..

[41] Lieu D (2010). Ultrasound physics and instrumentation for pathologists. Arch. Pathol. Lab. Med..

[42] Torrescano, G. (ed.). *Determination of Perimysium and Endomysium Thickness in Bovine, Ovine and Caprine Semimembranosus and Semitendinosus Muscles by Video Image Analysis* (2001).

[43] Dubost A, Micol D, Meunier B, Lethias C, Listrat A (2013). Relationships between structural characteristics of bovine intramuscular connective tissue assessed by image analysis and collagen and proteoglycan content. Meat Sci..

[44] Costello JR, Arif H, Kalb B, Martin DR, Maqbool M (2017). An Introduction to Medical Physics.

[45] Reimers K, Reimers CD, Wagner S, Paetzke I, Pongratz DE (1993). Skeletal muscle sonography: A correlative study of echogenicity and morphology. J. Ultrasound Med..

[46] Stock MS, Thompson BJ (2021). Echo intensity as an indicator of skeletal muscle quality: Applications, methodology, and future directions. Eur. J. Appl. Physiol..

[47] Nassiri DK, Nicholas D, Hill CR (1979). Attenuation of ultrasound in skeletal muscle. Ultrasonics.

[48] Pillen S, van Alfen N (2015). Muscle ultrasound from diagnostic tool to outcome measure—Quantification is the challenge. Muscle Nerve.

[49] Girts RM (2022). The influence of ultrasound image depth and gain on skeletal muscle echo intensity. Appl. Physiol. Nutr. Metabol..

[50] Schlegel W, Karger CP, Jäkel O (2018). Medizinische Physik.

