# Supplementary material for: Ex-vivo validation of spatial gain sonography for the quantification of echo intensity in fascicle-aligned ultrasound images in ten anatomical muscles in Bos taurus
Source: Sci Rep. 2024 Feb 15;14:3808. doi: 10.1038/s41598-024-53852-0 (PMC10869723; doi:10.1038/s41598-024-53852-0)
Supplement: Supplementary file 1 — Supplementary Information. [file 41598_2024_53852_MOESM1_ESM.docx]

# Supplementary Files

***Image Processing: Calculation of Pennation Angle and Fascicle Probe Angle***

1. **Pennation Angle**

The pennation angle was indicated manually on each ultrasound image and calculated using the custom-made *Python* script “QuantICUS” (Quantification of Intramuscular Connective Tissue with Ultrasound).

Supplementary Figure 1: Workflow for Ultrasound Image Angle Calculation in *Python*

Determining the pennation angle and ROI

- Superficial aponeurosis (yellow) and fascicle (red) are marked with two points each manually.


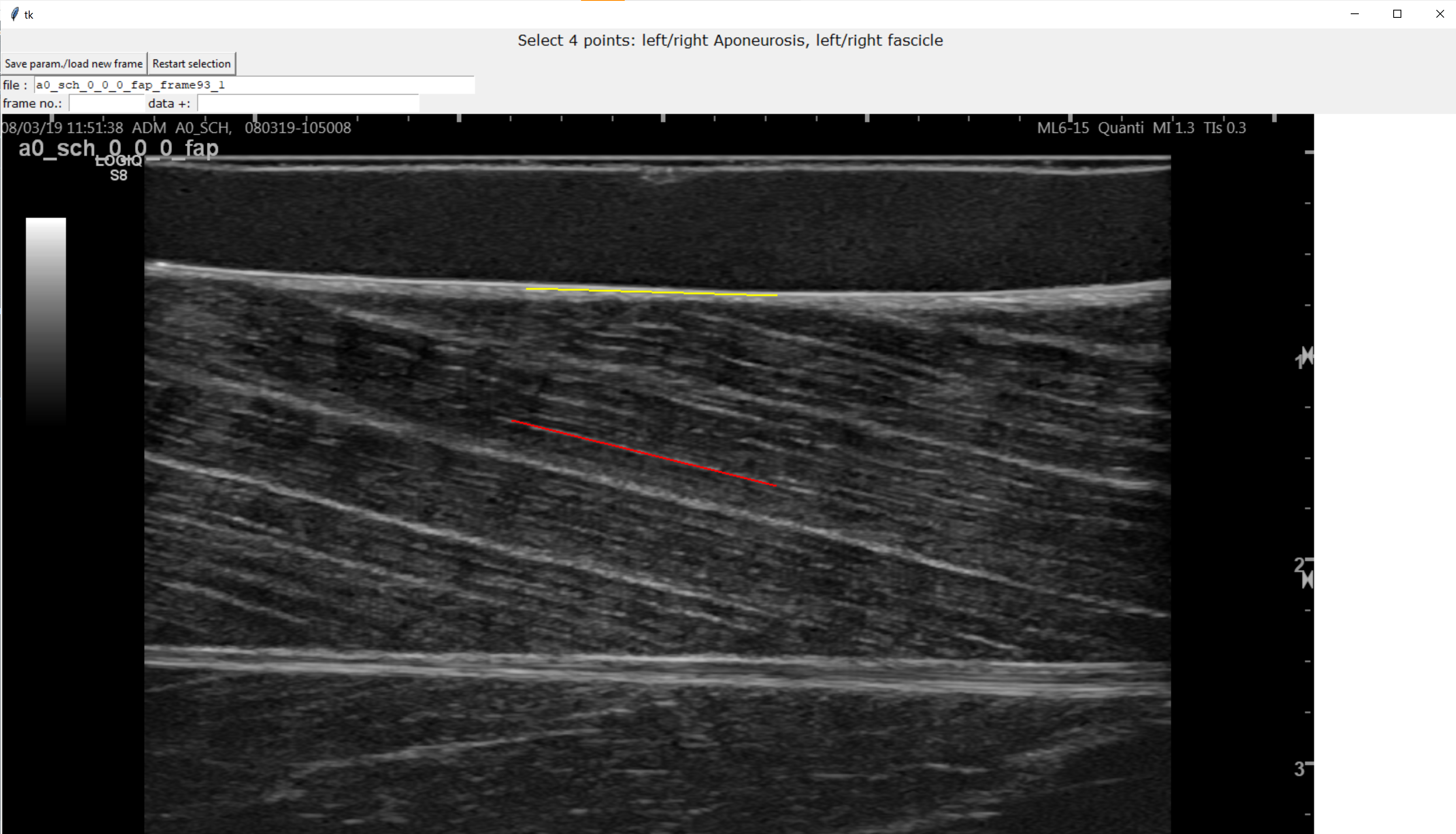


2. Setting region of interest

1. Setting aponeurosis and fascicle

- A rectangular region of interest (ROI) appears.


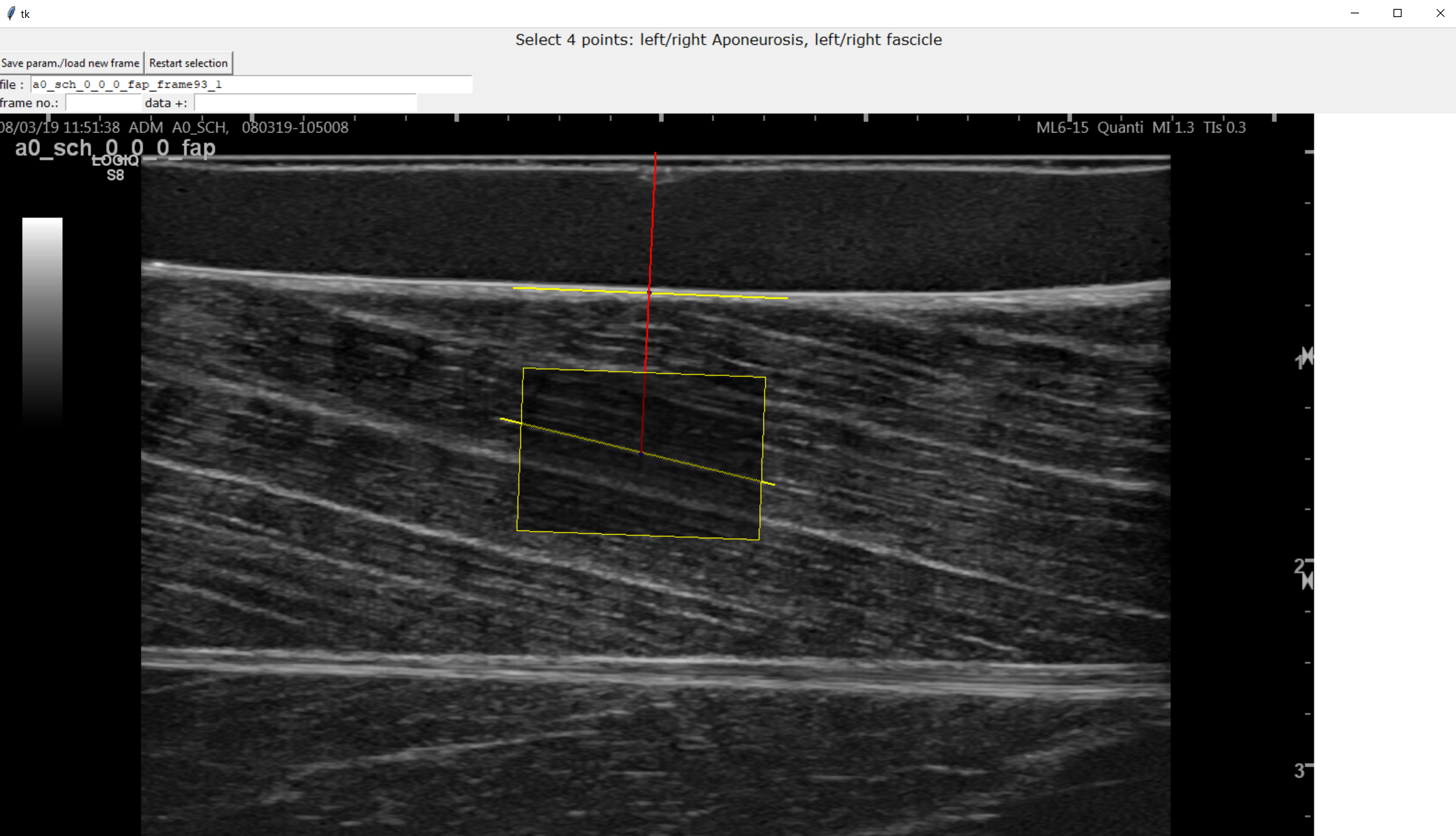


- The ROI is adjusted in the primary image to avoid blood vessels and fat accumulations if necessary.

3. Measuring angles and echo intensity

- In all subsequent scans of that muscle, the ROI is computed by the script according to the angulation in the respective images. Example 0° vs. 12° beam steering angle:


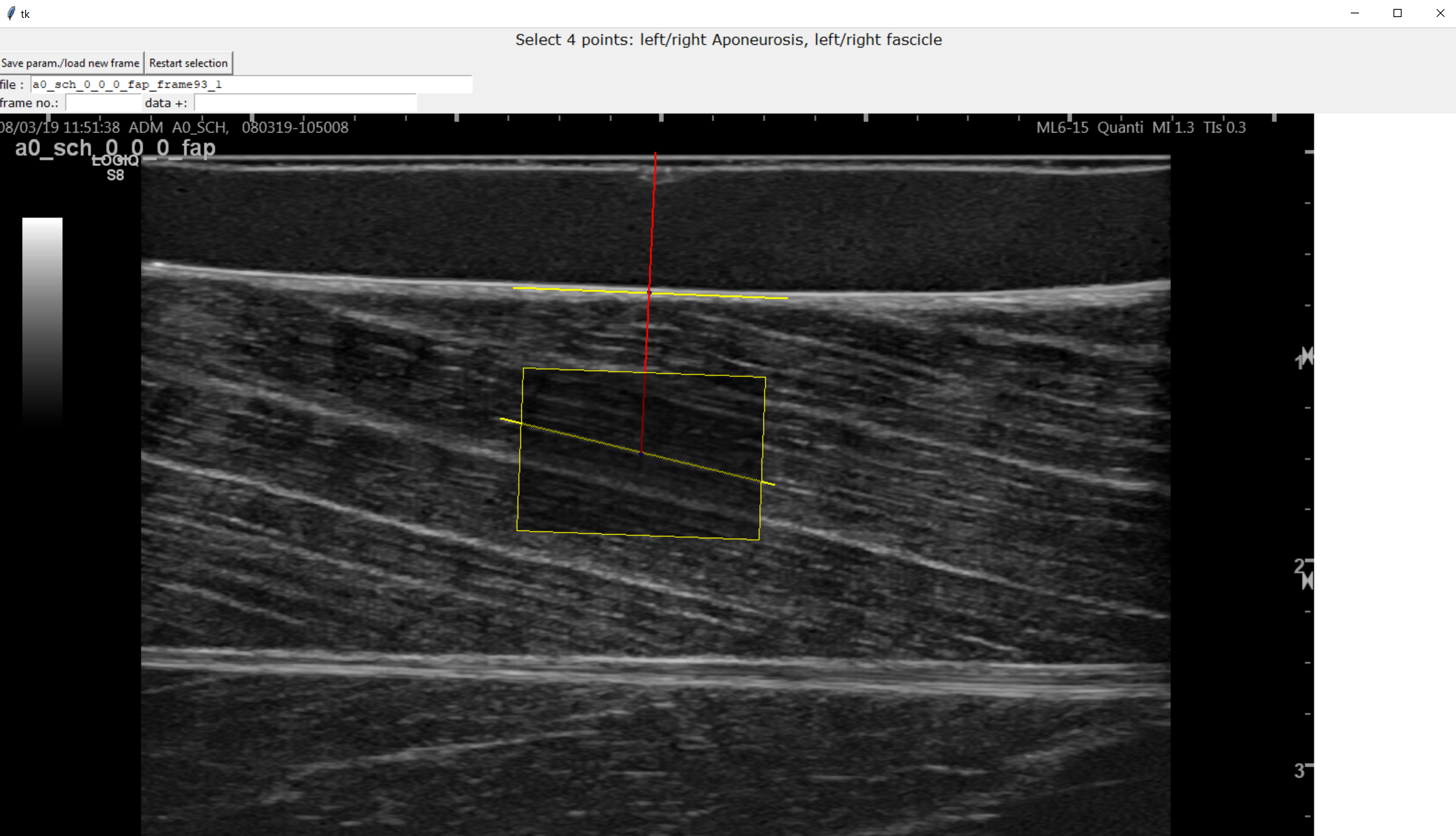


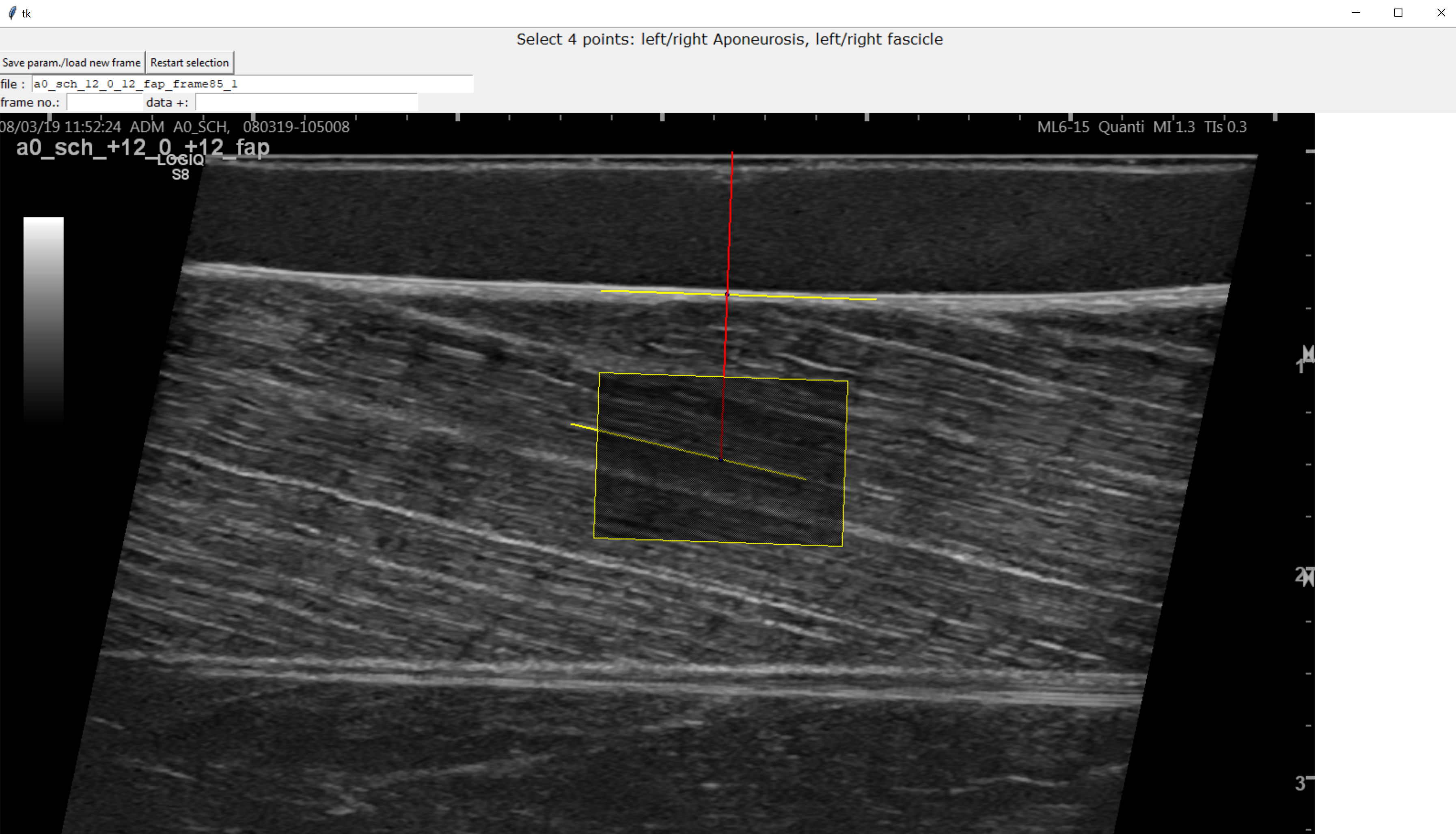


- The examiner does not move the ROI anymore, unless it is outside of the ultrasound image.
- Results: The aponeurosis probe angle and mean gray value inside the ROI are measured.
- The pennation angle is calculated.

1. **Fascicle Probe Angle**

The fascicle probe angle was calculated in *R* using the appropriate equation derived from the geometric outline as shown below. This image serves as an example and will differ slightly for each angle and gel pad configuration.


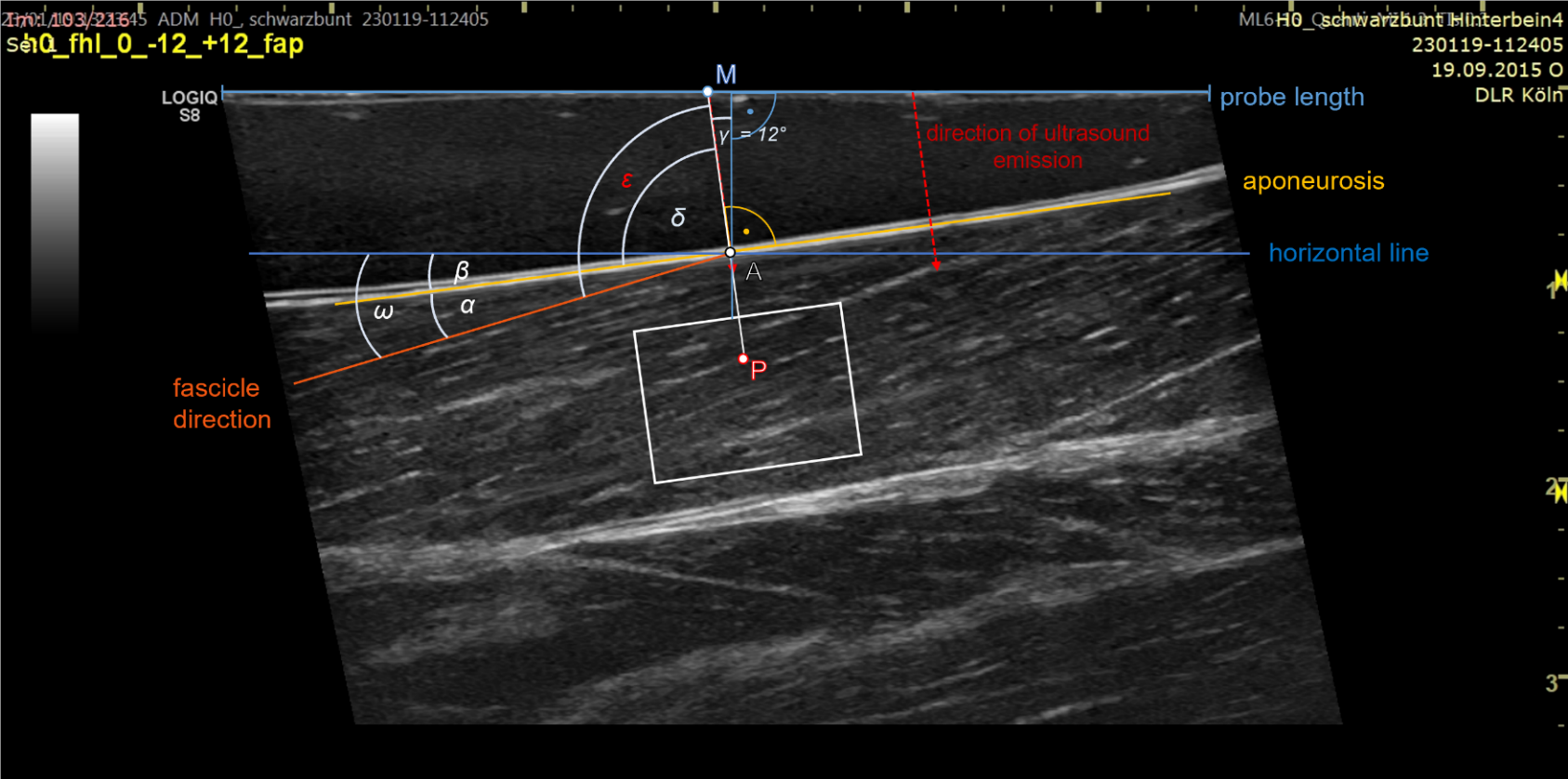


Supplementary Figure 2: Ultrasound image with angles α through ε used in image analysis.

| **Greek Letter in Suppl. Figure 2** | **Angle Name** | **Values or Equation** |
| --- | --- | --- |
| *α* | Pennation Angle | *α = ω - β* |
| *β* | Aponeurosis Probe Angle/  Measured Gel Pad Angle | see *α* |
| *γ* | Beam Steering Angle | +12/0/-12° |
| *δ* | Aponeurosis Insonation Angle | *δ = ε - \|α\|* |
| *ε* | Fascicle Insonation Angle | If *α>*0° (ω > β), then ε = 90° + ω - γ  *If α*<0° (ω < β), then ε = 90° - ω - γ |
| *ω* | Fascicle Probe Angle | see *ε* |
| *-* | Given Gel Pad Angle | +24/+12/0/-12/-24° |

Supplementary Table 1: Angle descriptions displayed in ultrasound image in Supplementary Figure 2 and their mathematical relationship.

***Supplementary diagrams***

| 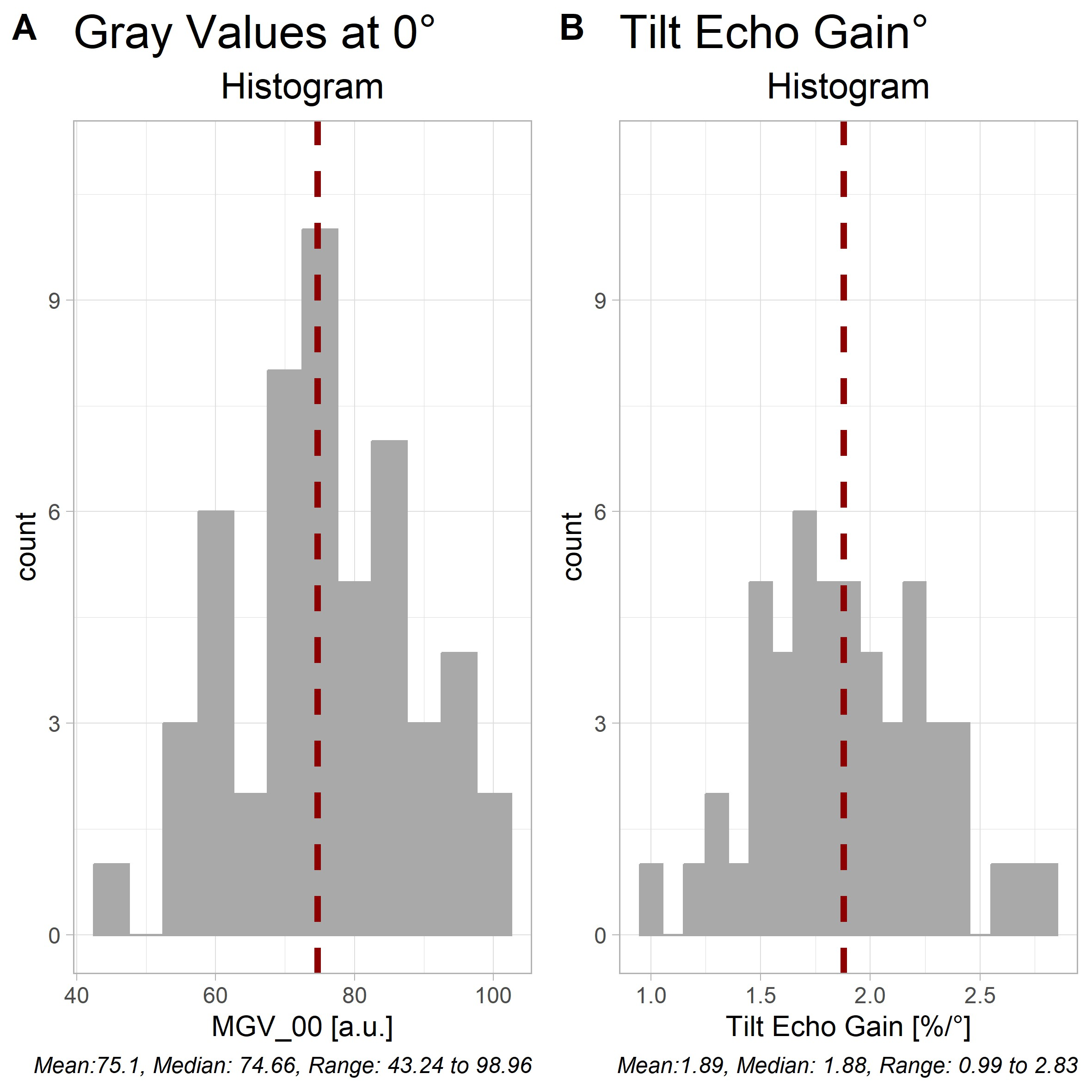 |
| --- |
| Supplementary Figure 3: Histograms showing the mean as a red line for MGV_00 (A) and TEG (B) of all analyzed muscles.  **A:** Mean gray value at FPA 0° ranged from 43.24 to 98.96, while the mean and most frequent value was 75.1.  **B:** TEG ranged from 0.99 to 2.83 across all muscles. The arithmetic mean was 1.89. |

| 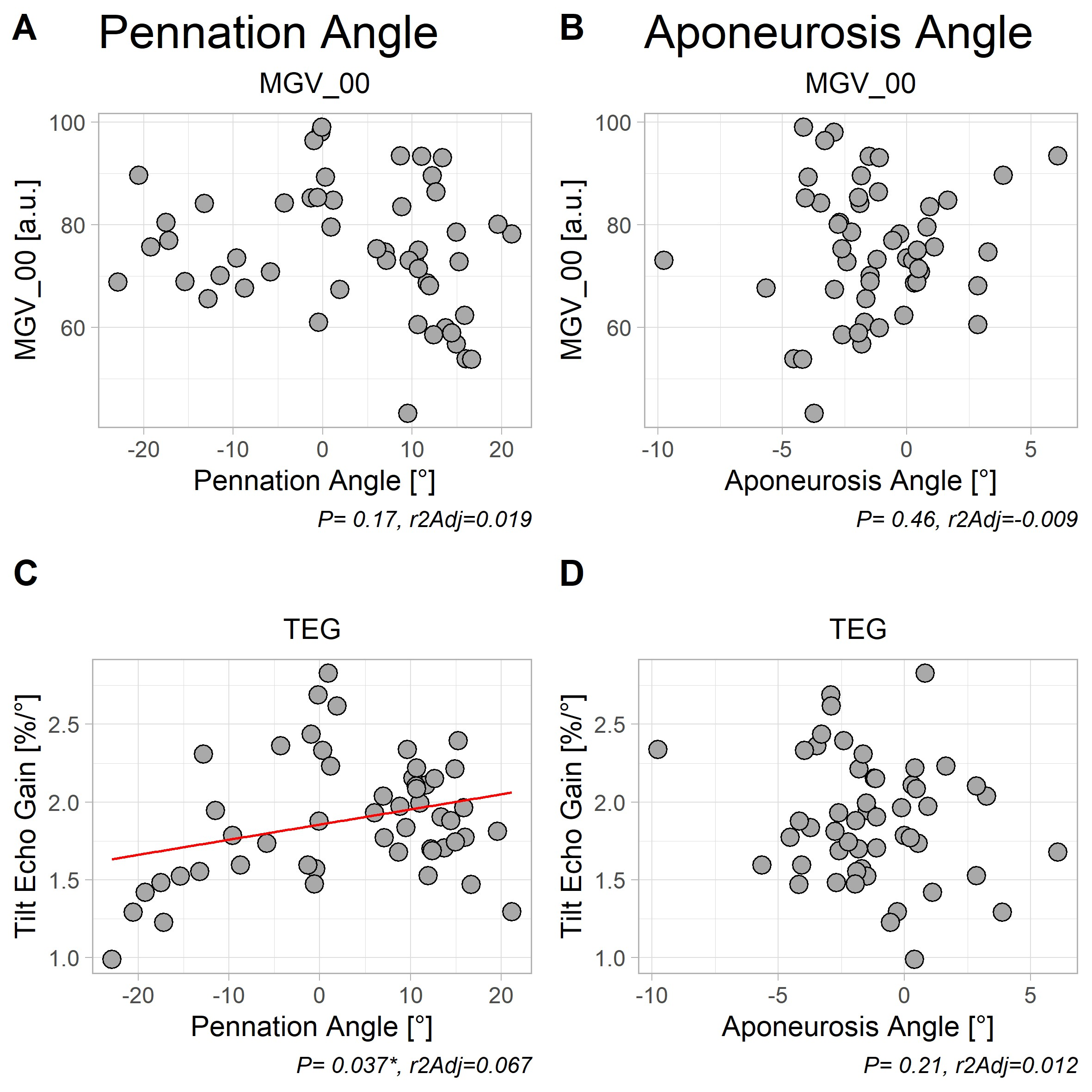 |
| --- |
| Supplementary Figure 4: Relationship of pennation and aponeurosis angle to MGV_00 and tilt echo gain (TEG).  **A/B:** Pennation angle and aponeurosis angle did not significantly affect MGV_00.  **C/D:** Pennation angle had a significant influence on TEG, however with a very weak correlation and aponeurosis angle had no significant influence on TEG. |
